# Effectiveness and Safety of Combination Therapy with Herbal Medicine and Growth Hormone Compared to Growth Hormone Monotherapy for Short Stature Children: A Systematic Review and Meta-Analysis

**DOI:** 10.1155/2022/5725258

**Published:** 2022-08-09

**Authors:** Soo Bo Shim, Hye Li Ahn, Hyun Hee Lee, Ju Ah Lee, Hye Lim Lee

**Affiliations:** ^1^Department of Paediatrics, College of Korean Medicine, Daejeon University, Daejeon, Republic of Korea; ^2^Hwa-pyeong Institute of Integrative Medicine, Incheon, Republic of Korea

## Abstract

**Introduction:**

Herbal medicines (HM) and growth hormones (GH) are widely used to treat short stature (SS) in children. This systematic review aimed to evaluate the effectiveness and safety of combination therapy with HM and GH (CHG) compared to those of GH monotherapy (GHM) in children with SS.

**Methods:**

We searched 17 electronic databases from inception to 1 April 2021. Only randomized controlled trials (RCTs) were included. Two authors independently performed the selection and quality assessment of the included studies using Cochrane Handbook criteria. Relative risk (RR) was used to measure dichotomous outcomes with a 95% confidence interval (CI). Mean difference (MD) or standard MD (SMD) was used to measure continuous outcomes with a 95% CI.

**Results:**

Seven RCTs involving 455 participants with SS were included. Standard deviations in height (MD = 0.31, 95% CI: 0.24–0.38, *p* < 0.00001), and insulin-like growth factor binding protein-3 (MD = 1.39, 95% CI: 0.93–1.85, *p* < 0.00001) were significantly higher in the CHG group than in the GHM group. Growth velocity (MD = 1.82, 95% CI: 1.34–2.31, *p* < 0.00001) and insulin-like growth factor-1 (MD = 61.85, 95% CI: 55.80–67.90, *p* < 0.00001) were significantly higher in the CHG group. Adverse events were significantly lower in the CHG group (risk ratio: 0.10, 95% CI: 0.02–0.54, *p* = 0.007). However, the level of evidence was low.

**Conclusions:**

CHG demonstrated significantly better efficacy than GHM for treating SS, with a low incidence of adverse events. However, since the level of evidence is low, methodologically standardized RCTs are required to verify these results.

## 1. Introduction

Individuals with short stature (SS) are two or more standard deviations (SDs) shorter than those with an average height of the same age and sex [1]. Approximately, 80% of individuals with SS have idiopathic SS (ISS), without identifiable deficiencies or disorders of growth hormone (GH) and endocrine, organ system, or genetic disorders [2]. Interest in height growth is expanding globally, and studies have reported that shorter height significantly impacts children's quality of life and depression scores [3, 4]. In particular, studies have reported that ISS can cause emotional issues in children and adolescents and result in socio-psychological and socio-economic effects [5, 6].

In Korea, recombinant human GH injection is a conventional treatment for ISS since the United States (US) Food and Drug Administration (FDA) approved the use of GH in 2003 [7]. Several studies have reported that administering GH in patients with ISS is effective in increasing the predicted adult height (PAH) and improving the growth velocity (GV) [8–10]. However, its long-term effects remain controversial [11].

There have been reports of certain side effects of GH, including elevated intracranial pressure, scoliosis, slipped capital femoral epiphysis, and type 2 diabetes [12]. It is recommended that side effects should be monitored, and fasting blood sugar and HbA1c levels should be measured every 3–6 months. In contrast, insulin-like growth factor-1 (IGF-1) and insulin-like growth factor binding protein-3 (IGFBP-3) should be tested every 6–12 months. Daily injections of GH and frequent follow-up blood tests are additional psychological burdens for children who undergo GH therapy, which often lasts for more than 2 years [12]. Furthermore, it is of concern that the efficacy decreases with prolonged treatment compared with that obtained during the first year of treatment. In Korea, survey results indicate low satisfaction with GH therapy (29.1%), and studies reveal that GH therapy does not positively impact children's quality of life and self-esteem [13, 14]. Due to various concerns, such as potential side effects and the fear of frequent injections among children and adolescents, complementary and alternative medical treatments are often sought to increase height growth [15]. Herbal medicine (HM) has been used to treat various diseases and is currently one of the most popular complementary and alternative medicines [16]. HM has been used for thousands of years in East Asia to treat growth disorders and SS. Previous studies used animal models to demonstrate that HM acted on different pathways from those targeted by GH by inducing the expression of IGF-1 and bone morphogenetic protein 2 and promoting longitudinal bone growth through cell proliferation in the epiphyseal plate [17, 18].

Systematic reviews on the treatment of SS primarily focused on the effects of GH [10, 19–21], and currently, investigations on the effects of HM are also being conducted [16, 22]. Several studies have been performed to determine the effects of combination therapy with HM and GH (CHG) for treating SS. However, currently, there is no review comparing the efficacy and safety of GH monotherapy (GHM) and CHG in patients with SS. Therefore, this study aimed to evaluate the effectiveness and safety of CHG compared to those of GHM in children with SS.

## 2. Methods

### 2.1. Study Protocol and Registration

The protocol of this systematic review and meta-analysis was registered on the Open Science Framework (OSF) platform, with the option to prospectively register a systematic review [23] (registration number: DOI 10.17605/OSF.IO/NMY5G). The protocol has been previously published [24].

### 2.2. Inclusion Criteria for Studies

#### 2.2.1. Types of Studies

Randomized controlled trials (RCTs) evaluating the effectiveness of GHM and CHG in treating SS were included.

#### 2.2.2. Participants

This study included children aged 0–18 years who exhibited decreased GV or were diagnosed with ISS according to the following diagnostic criteria: two or more SDs lower than the average height of an individual of the same age and sex, without identifiable deficiencies or disorders of GH.

#### 2.2.3. Types of Interventions

In this review, we only included studies that compared CHG and GHM. The presentations of HM, such as decoction, capsules, tablets, pills, powders, and extracts, were not restricted. In addition, only HM prescribed by traditional East Asian medicine doctors was included in the study.

#### 2.2.4. Outcomes

The primary outcome was an improvement in growth-related anthropometric indicators, such as PAH, GV, and SDs in height, and changes in growth-related hormones, such as IGF-1 and IGFBP-3. The secondary outcome was an improvement in height and weight. The incidence of adverse events (AE) during treatment was recorded.

### 2.3. Search Methods for the Identification of Studies

#### 2.3.1. Electronic Searches

We searched Medline, EMBASE, Central, CINAHL, AMED, and the East Asian databases, such as OASIS, KTKP, KISS, KoreaMed, KMbase, RISS, DBPIA, CNKI, Wanfangdata, CQVIP, CiNii, and J-stage. All studies available in the respective databases, from inception to 1 April 2021, were included in the search strategy.

#### 2.3.2. Search Strategy

The search terms and text words were: (“Short stature” [TIAB] OR “idiopathic short stature”[TIAB] OR “Growth disorder”[Mesh] OR “Dwarfism”[Mesh] OR “Failure to Thrive”[Mesh]) AND (“Herbal medicine”[Mesh] OR “Medicine, Korean Traditional”[Mesh] OR “Medicine, Chinese Traditional”[Mesh] OR “Medicine, Kampo”[Mesh] OR “Drug, Chinese Herbal”[Mesh] OR “Plant extract”[Mesh] OR “Plants, Medicinal”[Mesh] OR “traditional oriental medicine”[TIAB] OR “traditional Korean medicine”[TIAB] OR “traditional Chinese medicine”[TIAB] OR “kampo medicine”[TIAB] OR “herb^*∗*^”[TIAB]) AND (“Randomized Controlled Trial”[Publication Type] OR “Controlled clinical trial”[Publication Type] OR “randomized”[TIAB]). No language limitations were imposed. All details of the search strategy are described in the protocol article [24] and Supplementary Table 1.

### 2.4. Data Collection and Analysis

#### 2.4.1. Study Selection

**Figure 1:**
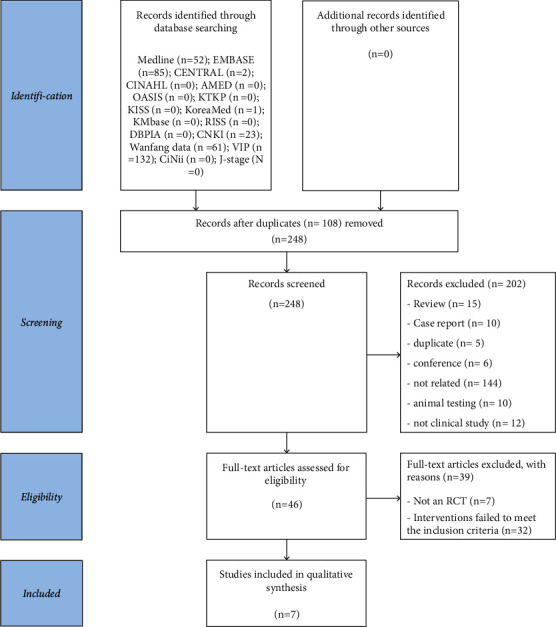
Flowchart showing the results of the literature search. AMED, Allied and Complementary Medicine Database; CENTRAL, Cochrane Central Register of Controlled Trials; CINAHL, Cumulative Index to Nursing and Allied Health Literature; CNKI, China National Knowledge Infrastructure; OASIS, Oriental Medicine Advanced Searching Integrated System; RISS, Research Information Service System; KISS, Korean Studies Information Service System; KTKP, Korean Traditional Knowledge Portal.

Two authors (SBS and JAL) independently performed the study selection. We excluded studies that did not fulfill the inclusion criteria (see Supplementary Table 2). Any disagreement was resolved through discussion and consensus with a third researcher (HLL). The selection process was recorded in the Preferred Reporting Items for Systematic Review and Meta-Analysis (PRISMA) flow diagram (Figure 1).

#### 2.4.2. Data Extraction and Management

Two authors (SBS and HLA) independently performed data extraction using a data extraction form in Microsoft Excel (Microsoft, Redmond, WA, USA). In case of discrepancies, we rechecked the extracted data and attempted to resolve them through discussion. The unresolved issues after the discussion were referred to a third reviewer (HHL) for arbitration.

We extracted the following items: data for study identification (last name of the first author, publication year, and country); details of the participants (age, sex, height, and height percentile), sample size, the details of the interventions (treatment group, control group, duration of the intervention, and composition of HM, if possible), outcome measures, results, and AE. When data were missing in the reports, we contacted the corresponding author of the RCT for missing information.

#### 2.4.3. Assessment of Risk of Bias in the Included Studies

Two researchers (SBS and HHL) assessed the risk of bias in the included studies according to the Cochrane Handbook criteria, version 5.2.0. We evaluated the following seven items: random sequence generation, allocation concealment, blinding of participants and personnel, blinding of outcome assessment, incomplete outcome data, selective reporting, and other bias as “Low risk,” “High risk”, and “Unclear risk” [25].

#### 2.4.4. Measures of Treatment Effect

We used relative risk (RR) to measure dichotomous outcomes and mean difference (MD) for continuous outcomes with a 95% confidence interval (CI). If the studies presented results on different scales, we used the standard MD (SMD) with a 95% CI.

#### 2.4.5. Assessment of Heterogeneity

Chi-squared and I^2^ tests were used to test for heterogeneity, and I^2^ > 50% indicated high heterogeneity. We performed meta-analyses using random- or fixed-effects models based on our analyses of the collected data. A fixed-effect model was used when no statistical heterogeneity was detected among the trials (I^2^ < 50%). In contrast, a random-effects model was used if methodological heterogeneity was detected.

#### 2.4.6. Data Synthesis

We conducted the review in accordance with the guidelines of the Cochrane Handbook for Systematic Reviews of Interventions. We performed analyses using Review Manager (RevMan) version 5.4 for Windows (The Nordic Cochrane Centre, The Cochrane Collaboration, Copenhagen, Denmark). We used GRADEpro software (McMaster University/Evidence Prime, Inc., Hamilton, Canada) to create a table summarising the findings. We assessed the primary and secondary outcomes, and *p*-values ≤0.025 were considered statistically significant.

#### 2.4.7. Assessment of Reporting Biases

Due to the small number of included studies, we did not generate funnel plots to detect reporting biases.

#### 2.4.8. Subgroup Analysis and Sensitivity Analyses

Due to the small number of included studies, subgroup analyses and sensitivity analyses to explore the sources of potential heterogeneity were not performed.

## 3. Results

### 3.1. Description of the Included Studies

#### 3.1.1. Results of the Search

We identified 356 studies by searching electronic databases (Figure 1). In the Korea and Japan databases, no studies met the inclusion criteria. After excluding 108 duplicates, 202 studies were screened by reading their titles and abstracts. A total of 46 studies were potentially relevant, and full texts were screened for further assessment. Finally, seven studies [26–32] were included in this review.

#### 3.1.2. General Characteristics of Included Studies

All seven RCTs [26–32] were conducted in China and published between 2015 and 2020; all were published in Chinese. The included studies involved 455 participants with ISS, and the sample sizes ranged from 20 to 45 participants. All details of the general characteristics of the included studies are presented in Supplementary Table 3.

#### 3.1.3. Risk of Bias in the Included Studies

**Figure 2:**
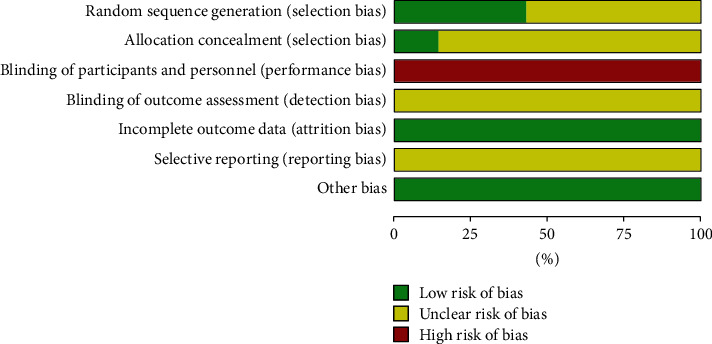
Risk of bias graph.

**Figure 3:**
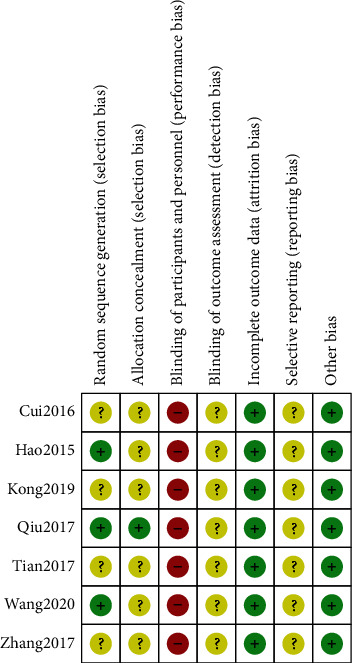
Risk of bias summary.

We observed that many RCTs had an unclear risk of bias in certain domains because we could not obtain information from the articles. Detailed information is shown in the “Risk of bias” graph and the “Risk of bias” summary (Figures 2 and 3). All details on the risk of bias in the included studies are summarised in Supplementary Table 3.

#### 3.1.4. Excluded Studies

We excluded 39 articles after careful examination of full-text copies. Common reasons for excluding studies were: no random allocation and HM monotherapy as the experimental intervention (see Supplementary Table 2).

### 3.2. Meta-Analysis Results

#### 3.2.1. Predicted Adult Height

None of the seven RCTs provided data on the PAH.

#### 3.2.2. Growth Velocity

**Figure 4:**
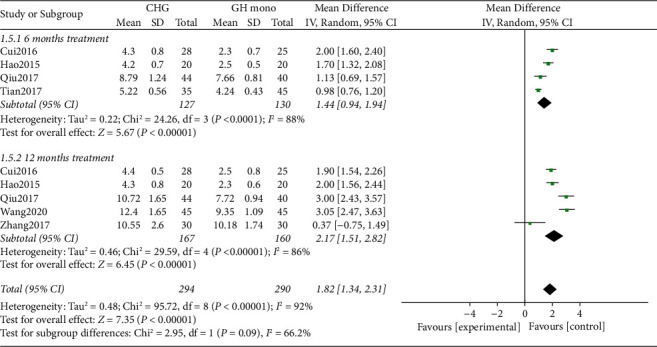
Forest plot comparing growth velocity between the CHG and GHM groups. CHG, combination therapy with herbal medicine and growth hormone; GHM, growth hormone monotherapy.

Six of the seven included RCTs [27–32] comparing the GV exhibited significant heterogeneity in the data (*p* < 0.00001, I^2^ = 92%, Figure 4). Owing to this significant heterogeneity in comparison, a random-effects model was applied. The results of the random-effects model analysis confirmed that the experimental group showed significant improvements in GV compared with the control groups (MD = 1.82, 95% CI: 1.34–2.31, *p* < 0.00001).

#### 3.2.3. SDs in Height

**Figure 5:**
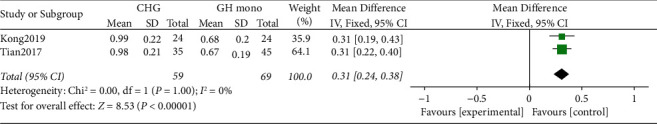
Forest plot comparing standard deviations in height between the CHG and GHM groups. CHG, combination therapy with herbal medicine and growth hormone; GHM, growth hormone monotherapy.

Two of the seven included RCTs [26,29] involving 128 participants were evaluated for the changes in SDs in height, with no significant heterogeneity in the data (*p* = 1.00, I^2^ = 0%; Figure 5). The results of the fixed-effects model analysis combined with effect sizes confirmed a significant difference between the experimental and control groups (MD = 0.31, 95% CI: 0.24–0.38, *p* < 0.00001), with the experimental group outperforming the control group.

#### 3.2.4. Insulin-Like Growth Factor 1

**Figure 6:**
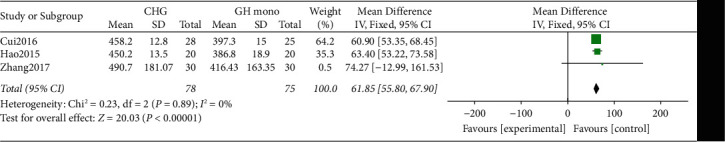
Forest plot comparing insulin-like growth factor 1 between the CHG and GHM groups. CHG, combination therapy with herbal medicine and growth hormone; GHM, growth hormone monotherapy.

Three of the seven included RCTs comparing IGF-1 showed no significant heterogeneity in the data [30–32] (*p* = 0.89, I^2^ = 0%; Figure 6). The results of the fixed-effects model analysis combined with effect sizes indicated a significant difference between the experimental and control groups (MD = 61.85, 95% CI: 55.80–67.90, *p* < 0.00001), with the experimental group outperforming the control group.

#### 3.2.5. Insulin-Like Growth Factor Binding Protein-3

**Figure 7:**
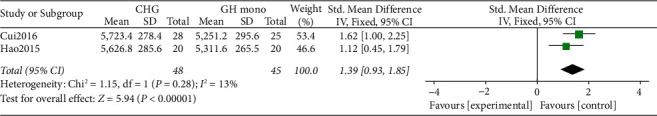
Forest plot comparing insulin-like growth factor binding protein-3 between the CHG and GHM groups. CHG, combination therapy with herbal medicine and growth hormone; GHM, growth hormone monotherapy.

Two of the seven included RCTs [30, 31] comparing IGFBP-3 showed no significant heterogeneity in the data (*p* = 0.28, I^2^ = 13%, Figure 7). The results of fixed-effects model analysis combined with effect sizes confirmed a significant difference between the experimental and control groups (SMD = 1.39, 95% CI: 0.93–1.85, *p* < 0.00001), with the experimental group outperforming the control group.

#### 3.2.6. Adverse Events

**Figure 8:**
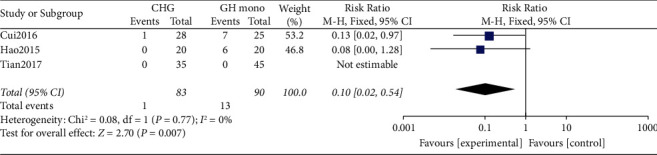
Forest plot comparing adverse events between the CHG and GHM groups. CHG, combination therapy with herbal medicine and growth hormone; GHM, growth hormone monotherapy.

Three of the seven included studies [29–31] comparing AEs showed no significant heterogeneity in the data (*p* = 0.77, I^2^ = 0%; Figure 8). The results of fixed-effects model analysis combined with effect sizes indicated a significant difference between the experimental and control groups (RR = 0.10, 95% CI: 0.02–0.54, *p* = 0.007). The experimental group had a significantly lower rate of AE than the control group.

### 3.3. Level of Evidence

The level of evidence is presented in Table 1. When comparing the CHG group with the GHM group, there was a “moderate” level of evidence for SDs in height [26, 29], weight (kg) [30,31], and IGFBP-3 (ng/mL) [30, 31] and “low” level of evidence for height (cm) [26, 27, 29–31], IGF-1 (ng/mL) [30–32], and AE [29–31]. The evidence level for GV (cm/year) [27–32] was “very low”. The low level of evidence was largely due to a high risk of bias, high heterogeneity, and overlap in the 95% CIs.

## 4. Discussion

This review provides a quantitative evaluation of the clinical efficacy and safety of CHG for treating SS by integrating outcomes from seven clinical RCTs involving 455 participants with ISS. The results of our analyses demonstrated that the efficacy of CHG was significantly better than that of GHM for treating SS. These results included significant improvements in GV, IGF-1, IGFBP-3, SDs in height, height, and weight, and a lower incidence rate of AEs in the CHG group than in the GHM group.

We could not perform meta-analyses for all our predefined outcomes because the data on PAH were not reported in any of the included RCTs. In Korea, PAH is often used as a primary indicator in height growth-related studies [22, 33]. Clinical RCTs using PAH as a major focus should be conducted in the future. If a meta-analysis on the change in PAH is conducted, the effect of CHG and GHM on height growth can be confirmed in more diverse ways.

The GV and SDs in height are critical evaluation indicators in height growth research and are used in many studies. However, due to the methodological limitations of clinical research, most studies did not reflect variations according to participants' age or sexual maturity. Therefore, heterogeneity may have occurred in the interpretation of the results. In future clinical RCTs, it is necessary to address heterogeneity by varying recruitment according to children's age or sexual maturity.

The results of these analyses indicated that CHG was effective in maintaining a low incidence of AE. Four cases of headache were reported in the GHM group and one in the CHG group. Two cases of obesity, three cases of redness and swelling at the injection site, and two cases of a decrease in serum T4 concentration were reported in the GHM group. The side effects of recombinant human GH therapy, currently used as a conventional treatment for ISS, may include injection site hematoma, pain, headache, scoliosis, glucose intolerance, and compromised cardiorespiratory functions [34, 35]. In two [30, 31] of the three studies [29–31] that reported AE, obesity, decrease in serum T4 concentration, and redness and swelling at the injection site were reported in the GHM group. In contrast, no AE was reported in the CHG group. Therefore, CHG is believed to mitigate the side effects; however, the clinical significance is limited.

### 4.1. Limitations

Overall, the evidence from the present review supports using CHG to treat children with SS. However, the RCTs included in this systematic review have several significant limitations.

First, all the research was conducted in China, which could be attributed to the widespread use of HM in Asian countries; however, it can be a potential bias factor. Explicit strategies, including databases from China, South Korea, and Japan, were designed to implement all available searches, but no search results were found. Furthermore, since our search was conducted until March 2021, further research related to our review may have been published, which could be included in future research.

Second, no negative results were reported and there may have been a reporting bias. Although publication bias could not be verified due to the small number of included studies, the fact that no negative results were reported may be considered a bias.

Third, the methodological quality of the included studies was low. None of the seven included studies reported blinding of participants and personnel, and only one study reported allocation concealment. In addition, no studies used the consolidated standards of reporting trials (CONSORT) checklist.

Fourth, there was a limitation in increasing the research heterogeneity and reducing reliability. The intervention dosage was not specified, the long-term follow-ups were not performed, and variables that significantly impacted results, such as participants' age or sexual maturity, were not controlled.

Finally, none of the included studies suggested the mechanisms underlying the synergistic effect exhibited by GH and HM. This makes it difficult to judge the clinical significance of the combination therapy.

### 4.2. Implications for Practice

The results of this study suggest that CHG has a positive effect in treating SS compared to GHM. As a viable and integrated approach to treating SS, CHG can be used as an effective complementary and alternative medical treatment option in combination with conventional GH therapy. Therefore, in clinical practice, more integrated treatment approaches are required to improve the effectiveness of treatment.

### 4.3. Implications for Research

The literature included in this systematic review had poor methodological quality, resulting in a reduced recommendation grade and level of evidence for systematic evaluation. Therefore, future clinical research should be conducted while considering the following factors:

1. In compliance with the CONSORT checklist, randomization, allocation concealment, and blinding should be described in detail.
2. Methodological standardization of RCT evaluation related to composition, dosage, and usage of HM therapy is required.
3. In research on childhood growth, the heterogeneity of results should be addressed by considering the participants' age and sexual maturity when recruiting.
4. In many countries, well-designed, large-scale, high-quality, multicentre RCTs are necessary for a more reliable research evaluation.

## 5. Conclusion

CHG provided significant improvements in GV and changes in SDs in height compared to GHM, maintaining a low incidence of AE. Therefore, we can assume that HM can help improve the effectiveness of GH and reduce the side effects. Despite these benefits, there are some limitations, such as the poor methodological quality of the analyzed studies and the high heterogeneity of the results. Furthermore, a definite mechanism through which GH and HM synergistically improve height growth has not yet been described. Before CHG is accepted as an evidence-based treatment option in clinical practice, more methodologically improved research data are required.

## Figures and Tables

**Table 1:** Summary of findings.

Patients or population: idiopathic short stature					
Intervention: CHG					
Comparison: GH monotherapy					
Outcomes	Anticipated absolute effects^*∗*^ (95% CI)		Relative effect (95% CI)	No. of participants (studies)	Certainty of the evidence (GRADE)
	Risk with GH monotherapy	Risk with CHG			
Height (cm)	The mean height (cm) was **0**	MD **3.32** (2.7–3.93) higher	—	420 (5 RCTs)	⊕⊕○○ LOW^a,b^
Change in height SDs	The mean change in height SDS was **0**	MD 0.31 (0.24–0.38) higher	—	128 (2 RCTs)	⊕⊕⊕○ MODERATE^a^
Weight (kg)	The mean weight (kg) was **0**	MD **2.47** (1.83–3.11) higher	—	186 (2 RCTs)	⊕⊕⊕○ MODERATE^a^
Growth velocity (cm/year)	The mean growth velocity (cm/year) was **0**	MD **1.82** (1.34–2.31) higher	—	584 (6 RCTs)	⊕○○○ VERY LOW^a,b,c^
IGF-1 (ng/mL)	The mean IGF-1 was **0**	MD **61.85** (55.8–67.9) higher	—	153 (3 RCTs)	⊕⊕○○ LOW^a,b^
IGFBP-3 (ng/ml)	—	SMD **1.39** (0.93–1.85) higher	—	93 (2 RCTs)	⊕⊕⊕○ MODERATE^a^
Adverse events	144 per 1 000	**14 per** 1 000 (3–78)	**RR** 0.10 (0.02–0.54)	173 (3 RCTs)	⊕⊕○○ LOW^a,b^

GRADE Working Group grades of evidence; High certainty: We are very confident that the true effect lies close to that of the estimate of the effect; Moderate certainty: We are moderately confident in the effect estimate. The true effect is likely to be close to the estimate of the effect, but there is a possibility that it is substantially different; Low certainty: Our confidence in the effect estimate is limited. The true effect may be substantially different from the estimate of the effect; Very low certainty: We have very little confidence in the effect estimate. The true effect is likely to be substantially different from the estimate of the effect. ^*∗*^The risk in the intervention group (and its 95% confidence interval) is based on the assumed risk in the comparison group and the relative effect of the intervention (and its 95% CI). ^a^In all RCTs, the blinding of participants and personnel was incomplete. ^b^Confidence intervals cross null effect. ^c^Heterogeneity is very high (I^2^ = 92%). CI, confidence interval; MD, mean difference; SDs, standard deviation; SMD, standardized mean difference; RR, risk ratio.

## Data Availability

The data used to support the findings of this study are included in the article as supplementary tables.

## Supplementary Materials

Supplementary Materials Supplementary Table 1. Details of search strategy; Supplementary Table 2. List of excluded studies; Supplementary Table 3. Characteristics of included studies.
