# Supplementary material for: Effectiveness and Safety of Combination Therapy with Herbal Medicine and Growth Hormone Compared to Growth Hormone Monotherapy for Short Stature Children: A Systematic Review and Meta-Analysis
Source: Evid Based Complement Alternat Med. 2022 Aug 9;2022:5725258. doi: 10.1155/2022/5725258 (PMC9381207; doi:10.1155/2022/5725258)
Supplement: Supplementary Materials — Supplementary Table 1. Details of search strategy; Supplementary Table 2. List of excluded studies; Supplementary Table 3. Characteristics of included studies. [file 5725258.f1.docx]

**Supplementary table 1. Details of search strategy**

| Database | Time span | Search strategy |
| --- | --- | --- |
| MEDLINE | to Apr 2021 | #1 Search (Short stature or idiopathic short stature)[Title/Abstract] OR (Growth disorder or Dwarfism or Failure to Thrive)[Mesh] #2 Search (Herbal medicine or Medicine, Korean Traditional or Medicine, Chinese Traditional or Medicine, Kampo or Drug, Chinese Herbal or Plant extract or Plants, Medicinal)[Mesh] OR (traditional oriental medicine or traditional korean medicine or traditional chinese medicine or kampo medicine or herb*)[Title/Abstract] #3 Search (Randomized Controlled Trial or Controlled clinical trial)[Publication Type] OR (randomized)[Title/Abstract] #4 #1 AND #2 AND #3 |
| EMBASE | to Apr 2021 | #1'short stature':ab,ti OR 'idiopathic short stature':ab,ti OR 'dwarfism':ab,ti OR 'growth disorder':ab,ti OR 'failure to thrive':ab,ti #2 'herbal medicine':ab,ti OR 'chinese medicine':ab,ti OR 'herb':ab,ti OR 'korean medicine':ab,ti OR 'kampo medicine (drug)':ab,ti OR 'plant extract':ab,ti #3 [controlled clinical trial]/lim OR [randomized controlled trial]/lim #4 #1 AND #2 AND #3 |
| CENTRAL | to Apr 2021 | #1 ("short-statured"):ti,ab,kw OR (idiopathic short stature):ti,ab,kw OR (dwarfism):ti,ab,kw OR (growth disorder):ti,ab,kw OR ("failure to thrive"):ti,ab,kw #2 (herbal medicine):ti,ab,kw OR (herb):ti,ab,kw OR ("traditional Chinese medicine"):ti,ab,kw OR (traditional korean medicine):ti,ab,kw OR (plant extract):ti,ab,kw #3 ("randomized controlled clinical trial"):pt OR ("controlled clinical trial"):pt #4 #1 AND #2 AND #3 |
| CINAHL | to Apr 2021 | #1 TI short stature OR TI idiopathic short stature OR TI dwarfism OR TI growth disorder OR TI failure to thrive  #2 TI herbal medicine OR TI traditional chinese medicine OR TI traditional korean medicine OR TI traditional oriental medicine OR TI kampo medicine OR TI herb  #3 TI randomized controlled trials OR TI controlled trial  #4 #1 AND #2 AND #3 |
| AMED | to Apr 2021 | (short stature or growth disorder) and (herbal medicine or herb) and (randomized controlled trial) |
| OASIS | to Apr 2021 | (short stature or growth disorder) and (herbal medicine or herb) and (randomized controlled trial) |
| KTKP | to Apr 2021 | (short stature or growth disorder) and (herbal medicine or herb) and (randomized controlled trial) |
| KISS | to Apr 2021 | (short stature or growth disorder) and (herbal medicine or herb) and (randomized controlled trial) |
| KoreaMed | to Apr 2021 | #1 ("short stature"[TIAB]) OR ("idiopathic short stature"[TIAB]) OR ("dwarfism"[MH]) OR ("growth disorder"[MH]) OR ("failure to thrive"[MH]) #2 ("herbal medicine"[TIAB]) OR ("traditional chinese medicine"[TIAB]) OR ("traditional korean medicine"[TIAB]) OR ("traditional oriental medicine"[TIAB]) OR ("kampo medicine"[TIAB]) OR ("herb[TIAB]) #3 "Randomized controlled trial"[TIAB] #4 #1 AND #2 AND #3 |
| KMBase | to Apr 2021 | (short stature or growth disorder) and (herbal medicine or herb) and (randomized controlled trial) |
| RISS | to Apr 2021 | (short stature or growth disorder) and (herbal medicine or herb) and (randomized controlled trial) |
| DBPIA | to Apr 2021 | (short stature or growth disorder) and (herbal medicine or herb) and (randomized controlled trial) |
| China Network Knowledge Infrastructure (CNKI) | to Apr 2021 | Search strategy in Chinese. #1 Title/Abstract=(short stature or idiopathic short stature or Dwarfism or growth disorder) #2 Title/Abstract=(Herbal medicine or traditional chinese medicine or decoction or pill or powders) #3 Text word=(random or controlled) #4 #1 AND #2 AND #3 |
| Wanfang data | to Apr 2021 | Search strategy in Chinese. #1 Title/Abstract=(short stature or idiopathic short stature or Dwarfism or growth disorder) #2 Title/Abstract=(Herbal medicine or traditional chinese medicine or decoction or pill or powders) #3 Text word=(random or controlled) #4 #1 AND #2 AND #3 |
| Chongqing VIP (CQVIP) | to Apr 2021 | Search strategy in Chinese. #1 Title/Abstract=(short stature or idiopathic short stature or Dwarfism or growth disorder) #2 Title/Abstract=(Herbal medicine or traditional chinese medicine or decoction or pill or powders) #3 Text word=(random or controlled) #4 #1 AND #2 AND #3 |
| CiNii | to Apr 2021 | (short stature or growth disorder) and (herbal medicine or herb) and (randomized controlled trial) |
| J-stage | to Apr 2021 | (short stature or growth disorder) and (herbal medicine or herb) and (randomized controlled trial) |

**Supplementary table 2. List of excluded studies**

| Study ID | Reason for exclusion |
| --- | --- |
| Chen1996 | not randomized controlled trial |
| Yan1998 | not randomized controlled trial |
| Zhang1998 | not randomized controlled trial |
| Yan1998 | not randomized controlled trial |
| Gu1999 | not randomized controlled trial |
| Lu2009 | herbal medicine + Lysine supplement versus Lysine supplement |
| Chai2000 | Three arms (herbal medicine + clonidine HCl, herbal medicine, clonidine HCl |
| Chen2010 | herbal medicine + Lysine supplement versus Lysine supplement |
| Hu2012 | Three arms (herbal medicine, rhGH, control) |
| Liu2012 | Three arms (herbal medicine + acupoint massage, rhGH, control) |
| Yu2012 | herbal medicine versus control |
| Hou2013 | not randomized controlled trial |
| Hu2013 | not randomized controlled trial |
| Du2013 | Herbal medicine versus rhGH |
| Li2013 | Herbal medicine versus control |
| Lv2013 | Four arms (herbal medicine + exercise, herbal medicine, exercise, control) |
| Feng2013 | Four arms (herbal medicine, acupoint massage, ear-acupuncture, control) |
| Wang2013 | not randomized controlled trial |
| Wang2014 | not randomized controlled trial |
| Wang2014 | herbal medicine versus control |
| Feng2014 | Four arms (herbal medicine, acupoint massage, ear-acupuncture, control) |
| Li2014 | herbal medicine versus Zinc supplement |
| Bi2014 | herbal medicine + acupuncture versus Zinc supplement |
| Xu2015 | not randomized controlled trial |
| Zhang2015 | herbal medicine versus Zinc supplement |
| Ye2015 | herbal medicine versus another herbal medicine |
| Zhu2015 | Three arms (Herbal medicine, rhGH, control |
| Yu2016 | herbal medicine + Zinc supplement versus Zinc supplement |
| Hong2016 | herbal medicine + Zinc supplement versus Zinc supplement |
| Li2017 | herbal medicine + Zinc supplement versus herbal medicine |
| Su2017 | herbal medicine versus Zinc supplement |
| Sun2017 | not randomized controlled trial |
| Sun2017 | herbal medicine versus control |
| Zhang2017 | not randomized controlled trial |
| Chen2018 | not randomized controlled trial |
| Zhang2018 | not randomized controlled trial |
| Cai2020 | herbal medicine + acupoint sticking therapy versus supplement |
| Jiang2020 | Herbal medicine versus rhGH |
| Wang2020 | herbal medicine versus exercise |

**Supplementary table 3. Characteristics of included studies**

| Cui2016 |  |  |
| --- | --- | --- |
| Methods | Randomised clinical trial, China  Parallel group design | |
| Participants | 53 participants diagnosed with idiopathic short stature according to chinese clinical pediatrics.  Male:female: not reported.  age: 6 to 13  Exclusion criteria: Hypothyroidism, Chronic systemic diseases, Osteodysplasia, Precocious puberty, Growth hormone difficiency (GH < 10 ug/L), Turner syndrome | |
| Interventions | Experimental Intervention: TCM, twice a day, po, & rhGH, 0.15~0.18 IU/kg/d, once daily, subcutaneous injection, 12 months (n=28)  Control Intervention: rhGH, 0.15~0.18 IU/kg/d, once daily, subcutaneous injection, 12 months (n=25)  Post-treatment follow-up: no follow-up | |
| Outcomes | Height, Weight (after 6, 12 months treatment)  Bone age changes, Growth velocity (0~6 months, 7~12 months of treatment)  IGF-1, IGFBP-3, ALP  Safety: LFT, TFT, Glucose, KFT | |
| Notes | Study dates: Mar 2012 to Oct 2015  Funding information: author did not provide any information on clinical study support or sponsorship.  TCM pattern: not reported.  Prescription form: Decoction  Prescription components: Ginseng Radix, Glycyrrhizae Radix et Rhizoma, Citri Unshius Pericarpium, Atractylodis Rhizoma Alba, Poria Sclerotium, Dioscoreae Rhizoma. Hordei Fructus Germinatus, Atractylodis Rhizoma, Oryzae Fructus Germinatus, Epimedii Herba | |
| **Risk of bias** | | |
| Bias | Author's judgement | Support for judgement |
| Random sequence generation (selection bias) | Unclear risk | Method of generating random number not reported |
| Allocation concealment (selection bias) | Unclear risk | Not reported |
| Blinding of participants and personnel (performance bias)  All outcomes | High risk | Author did not report that they blinded participants and personnel, and the administration route was different between groups, which can easily unseal the blinding |
| Blinding of outcome assessment (detection bias)  All outcomes | Unclear risk | Not reported |
| Incomplete outcome data (attrition bias)  All outcomes | Low risk | No missing data |
| Selective reporting  (reporting bias) | Unclear risk | No protocol was available |
| Other bias | Low risk | No other concerns identified |
|  |  |  |
| Qiu2017 |  |  |
| Methods | Randomised clinical trial, China  Parallel group design | |
| Participants | 84 participants diagnosed with idiopathic short stature with spleen and kidney deficiency type according to chinese clinical pediatrics.  Male:female: 41:43  age: 7 to 11  Exclusion criteria: Hypothyroidism, Precocious puberty, Brain tumor, Congenital heart disease, BA-CA > 1year, Growth hormone disorder (GH < 10 ng/mL or GH < 5ng/mL) | |
| Interventions | Experimental Intervention: TCM, once daily, 1 day rest after 6 days po, & rhGH, 0.1U/kg/d, once daily, 1 day rest after 6 days subcutaneous injection, 12 months (n=44)  Control Intervention: rhGH, 0.1U/kg/d, once daily, 1 day rest after 6 days subcutaneous injection, 12 months (n=40)  Post-treatment follow-up: no follow-up | |
| Outcomes | Bone age, Height, Growth velocity (3, 6, 12 months), TCM syndrome integral score (major, minor; 3, 5, 12 months)  Safety: LFT, TFT, Glucose | |
| Notes | Study dates: Mar 2014 to Feb 2016  Funding information: author did not provide any information on clinical study support or sponsorship.  TCM pattern: not reported.  Prescription form: Granules  Prescription components: Codonopsitis Radix 10g, Astragali Radix 10g, Atractylodis Rhizoma Alba 10g, Poria Sclerotium 10g, Dioscoreae Rhizoma 10g, Corni Fructus 6g, Rehmanniae Radix Preparata 10g, Psoraleae Semen 10g, Glycyrrhizae Radix et Rhizoma 6g | |
| **Risk of bias** | | |
| Bias | Author's judgement | Support for judgement |
| Random sequence generation (selection bias) | Low risk | Random number table used |
| Allocation concealment (selection bias) | Low risk | Centralised allocation provided using computer-generated code. |
| Blinding of participants and personnel (performance bias)  All outcomes | High risk | Author did not report that they blinded participants and personnel, and the administration route was different between groups, which can easily unseal the blinding |
| Blinding of outcome assessment (detection bias)  All outcomes | Unclear risk | Not reported |
| Incomplete outcome data (attrition bias)  All outcomes | Low risk | No missing data |
| Selective reporting  (reporting bias) | Unclear risk | No protocol was available |
| Other bias | Low risk | No other concerns identified |
|  |  |  |
| Tian2017 |  |  |
| Methods | Randomised clinical trial, China  Parallel group design | |
| Participants | 80 participants diagnosed with idiopathic short stature according to chinese clinical pediatrics. Male:female: 45:35  Mean age: 6.50±1.07 (Experimental group), 6.45±1.09 (Control group)  Exclusion criteria: Endocrinological deficiency, Chronic systemic disease, Congenital malformation, metabolic syndrome, chromosomal disease, hepatitis B, Tumor, Family history of Tumor, severe psychological disruption, puberty period | |
| Interventions | Experimental Intervention: TCM, twice a day po & rhGH, 0.15IU/kg/d, once daily subcutaneous injection, 6 months (n=35)  Control Intervention: rhGH, 0.15IU/kg/d, once daily subcutaneous injection, 6 months (n=45)  Post-treatment follow-up: no follow-up | |
| Outcomes | Height, Weight, IGF-1, IGFBP-3 (3, 6 months)  Growth velocity, Bone age (6 months)  Adverse events | |
| Notes | Study dates: Aug 2015 to Oct 2016  Funding information: author did not provide any information on clinical study support or sponsorship.  TCM pattern: spleen deficiency, kidney deficiency, dual deficiency of the spleen-kidney Prescription form: Granules  Prescription components:  Spleen deficiency: Sagoonja-Tang: Pseudostellariae Radix 10g, Astragali Radix10g, Atractylodis Rhizoma Alba10g, Poria Sclerotium10g, Glycyrrhizae Radix et Rhizoma3g  Kidney deficiency: Bosin-Jihuang-Hwan: Cervi Cornus Colla 5g, Dioscoreae Rhizoma10g, Rehmanniae Radix Preparata10g, Corni Fructus5g, Eucommiae Cortex10g, Achyranthis Radix5g, Poria Sclerotium10, Moutan Radicis Cortex5g, Alismatis Rhizoma5g  Dual deficiency of the spleen-kidney: Sagoonja-Tang with Bosin-Jihuang-Hwan: Pseudostellariae Radix 10g, Atractylodis Rhizoma Alba10g, Poria Sclerotium10g, Dioscoreae Rhizoma10g, Glycyrrhizae Radix et Rhizoma3g, Rehmanniae Radix Preparata10g, Corni Fructus5g, Eucommiae Cortex10g, Cervi Cornus Colla5g, Alismatis Rhizoma5g, Achyranthis Radix5g, Poria Sclerotium10, Moutan Radicis Cortex5g | |
| **Risk of bias** | | |
| Bias | Author's judgement | Support for judgement |
| Random sequence generation (selection bias) | Unclear risk | Method of generating random number not described |
| Allocation concealment (selection bias) | Unclear risk | Not reported |
| Blinding of participants and personnel (performance bias)  All outcomes | High risk | Author did not report that they blinded participants and personnel, and the administration route was different between groups, which can easily unseal the blinding |
| Blinding of outcome assessment (detection bias)  All outcomes | Unclear risk | Not reported |
| Incomplete outcome data (attrition bias)  All outcomes | Low risk | No missing data |
| Selective reporting  (reporting bias) | Unclear risk | No protocol was available |
| Other bias | Low risk | No other concerns identified |
|  |  |  |
| Zhang2017 |  |  |
| Methods | Randomised clinical trial, China  Parallel group design | |
| Participants | 60 participants diagnosed with idiopathic short stature according to chinese clinical pediatrics and 2008 chinese clinical guideline for short stature children.  Male:female: 32:28  Mean age: 10.26±2.62 (Experimental group), 10.75±2.78 (Control group)  Exclusion criteria: GH < 10ug/L, already inject rhGH, severe psychological disruption, systemic diseases, chronic wasting diseases such as tumor, Tuberculosis | |
| Interventions | Experimental Intervention: TCM, 150ml, twice a day po, 2 months & rhGH, 0.15IU/kg/d, once daily subcutaneous injection, 10 months (n=30). total 12 months  Control Intervention: rhGH, 0.15IU/kg/d, once daily subcutaneous injection, 12 months (n=30)  Post-treatment follow-up: no follow-up | |
| Outcomes | Height, Growth velocity, IGF-1, Vitamin D, effective rate  Safety: LFT, Glucose, TFT | |
| Notes | Study dates: Jun 2014 to Jun 2016  Funding information: author did not provide any information on clinical study support or sponsorship.  TCM pattern: liver blood deficiency, heart blood deficiency, liver depression and spleen deficiency  Priscription form: Decoction  Prescription components:  Bacis components: Pseudostellariae Radix10g, Astragali Radix10g, Atractylodis Rhizoma Alba10g, Dioscoreae Rhizoma10g, Poria Sclerotium10g, Rehmanniae Radix Preparata10g, Testudinis Chinemis Plastrum et Carapax 10g, Cervi Cornus Colla10g, Corni Fructus10g, Psoraleae Semen 10g, Citri Unshius Pericarpium 5g, Alismatis Rhizoma5g, Glycyrrhizae Radix et Rhizoma3g;  Liver blood deficiency: add Lycii Fructus 10g, Acanthopanacis Cortex 5g, Loranchi Ramulus Et Folium 10g;  Heart blood deficiency: add Acanthopanacis Cortex 5g, Polygalae Radix 10g, Acori Graminei Rhizoma 5g;  Liver depression and spleen deficiency: add Bupleuri Radix 5g, Meliae Fructus 5g, Corydalis Tuber 5g | |
| **Risk of bias** | | |
| Bias | Author's judgement | Support for judgement |
| Random sequence generation (selection bias) | Unclear risk | Method of generating random numver not described |
| Allocation concealment (selection bias) | Unclear risk | Not reported |
| Blinding of participants and personnel (performance bias)  All outcomes | High risk | Author did not report that they blinded participants and personnel, and the administration route was different between groups, which can easily unseal the blinding |
| Blinding of outcome assessment (detection bias)  All outcomes | Unclear risk | Not reported |
| Incomplete outcome data (attrition bias)  All outcomes | Low risk | No missing data |
| Selective reporting  (reporting bias) | Unclear risk | No protocol was available |
| Other bias | Low risk | No other concerns identified |
|  |  |  |
| Kong2019 |  |  |
| Methods | Randomised clinical trial, China  Parallel group design | |
| Participants | 48 participants diagnosed with idiopathic short stature according to chinese clinical pediatrics.  Male:female: 26:22  Mean age: 6.46±1.10 (Experimental group), 6.52±1.08 (Control group)  Exclusion criteria: Endocrinological diseases, Congenital malformation, metabolic syndrome, Chromosomal diseases. | |
| Interventions | Experimental Intervention: TCM, twice a day po & rhGH, 0.15IU/kg/d, once daily subcutaneous injection, 6 months (n=24)  Control Intervention: rhGH, 0.15IU/kg/d, once daily subcutaneous injection, 6 months (n=24)  Post-treatment follow-up: no follow-up | |
| Outcomes | Height, Weight, IGF-1, IGFBP-3, respiratory and digestive diseases rate | |
| Notes | Study dates: Sep 2016 to Sep 2017  Funding information: author did not provide any information on clinical study support or sponsorship.  TCM pattern: spleen deficiency, kidney deficiency, dual deficiency of the spleen-kidney  Prescription form: Decoction  Prescription components:  Spleen deficiency: Sagoonja-Tang: Pseudostellariae Radix, Atractylodis Rhizoma Alba, Astragali Radix each 10g, Poria Sclerotium 8g, Glycyrrhizae Radix et Rhizoma 5g Kidney deficiency: Bosin-Jihuang-Hwan: Dioscoreae Rhizoma, Rehmanniae Radix Preparata, Poria Sclerotium, Eucommiae Cortex each 10g, Cervi Cornus Colla, Corni Fructus, Achyranthis Radix, Moutan Radicis Cortex, Alismatis Rhizoma each 5g Dual deficiency of the spleen-kidney: Sagoonja-Tang with Bosin-Jihuang-Hwan: Pseudostellariae Radix, Atractylodis Rhizoma Alba, Poria Sclerotium, Dioscoreae Rhizoma each 10g, Rehmanniae Radix Preparata, Eucommiae Cortex, Poria Sclerotium each 12g, Corni Fructus, Cervi Cornus Colla, Alismatis Rhizoma, Achyranthis Radix each 5g, Moutan Radicis Cortex, Glycyrrhizae Radix et Rhizoma each 3g | |
| **Risk of bias** | | |
| Bias | Author's judgement | Support for judgement |
| Random sequence generation (selection bias) | Unclear risk | Method of generating random numver not described |
| Allocation concealment (selection bias) | Unclear risk | Not reported |
| Blinding of participants and personnel (performance bias)  All outcomes | High risk | Author did not report that they blinded participants and personnel, and the administration route was different between groups, which can easily unseal the blinding |
| Blinding of outcome assessment (detection bias)  All outcomes | Unclear risk | Not reported |
| Incomplete outcome data (attrition bias)  All outcomes | Low risk | No missing data |
| Selective reporting  (reporting bias) | Unclear risk | No protocol was available |
| Other bias | Low risk | No other concerns identified |
|  |  |  |
| Wang2020 |  |  |
| Methods | Randomised clinical trial, China  Parallel group design | |
| Participants | 90 participants diagnosed with idiopathic short stature according to chinese clinical pediatrics and 2008 chinese clinical guideline for short stature children.  Male:female: 32:58  Mean age: 8.7±1.5 (Experimental group), 9.1±1.3 (Control group)  Exclusion criteria: GH < 10ug/L, already inject rhGH, severe psychological disruption, systemic diseases, chronic wasting diseases such as tumor, Tuberculosis | |
| Interventions | Experimental Intervention: TCM, twice a day po & rhGH, 0.15~0.20 U/kg-1, once daily subcutaneous injection, 12 months (n=45)  Control Intervention: rhGH, 0.15~0.20U/kg-1, once daily subcutaneous injection, 12 months (n=45)  Post-treatment follow-up: no follow-up | |
| Outcomes | Height, Growth velocity, Bone age | |
| Notes | Study dates: Jan 2016 to Dec 2019  Funding information: author did not provide any information on clinical study support or sponsorship.  TCM pattern: not reported.  Prescription form: Granules  Prescription components: Pinelliae Tuber, Poria Sclerotium, Pseudostellariae Radix, Dioscoreae Rhizoma, Atractylodis Rhizoma Alba, Citrii Unshius Rericarpium, Thujae Semen, Glycyrrhizae Radix et Rhizoma | |
| **Risk of bias** | | |
| Bias | Author's judgement | Support for judgement |
| Random sequence generation (selection bias) | Low risk | Random number table used |
| Allocation concealment (selection bias) | Unclear risk | Not reported |
| Blinding of participants and personnel (performance bias)  All outcomes | High risk | Author did not report that they blinded participants and personnel, and the administration route was different between groups, which can easily unseal the blinding |
| Blinding of outcome assessment (detection bias)  All outcomes | Unclear risk | Not reported |
| Incomplete outcome data (attrition bias)  All outcomes | Low risk | No missing data |
| Selective reporting  (reporting bias) | Unclear risk | No protocol was available |
| Other bias | Low risk | No other concerns identified |
|  |  |  |
| Hao2015 |  |  |
| Methods | Randomised clinical trial, China  Parallel group design | |
| Participants | 40 participants diagnosed with idiopathic short stature according to clinical pediatrics.  Male:female: 21:19  Mean age: 8.8±1.6 (Experimental group), 8.7±1.5 (Control group)  Exclusion criteria: GH < 10ug/L, Hypothyroidism, bone malformation, chronic systemic diseases, precocious puberty, Turner syndrome | |
| Interventions | Experimental Intervention: TCM, twice a day po & rhGH, 0.15~0.18 U/kg/d, once daily subcutaneous injection, 12 months (n=20)  Control Intervention: rhGH, 0.15~0.18U/kg/d, once daily subcutaneous injection, 12 months (n=20)  Post-treatment follow-up: no follow-up | |
| Outcomes | Height, Weight (0~6 months, 7~12 months),  Growth velocity, Bone age, IGF-1, IGFBP-3, ALP, Adverse events | |
| Notes | Study dates: Jan 2016 to Dec 2019  Funding information: author did not provide any information on clinical study support or sponsorship.  TCM pattern: not reported  Prescription form: Decoction  Prescription components: Ziziphi Fructus, Astragali Radix, Spatholobi Caulis, Codonopsis Pilosulae Radix, Poria Sclerotium, Glycyrrhizae Radix et Rhizoma, Rehmanniae Radix Preparata, Polygoni Multiflori Radix | |
| **Risk of bias** | | |
| Bias | Author's judgement | Support for judgement |
| Random sequence generation (selection bias) | Low risk | Random number table used |
| Allocation concealment (selection bias) | Unclear risk | Not reported |
| Blinding of participants and personnel (performance bias)  All outcomes | High risk | Author did not report that they blinded participants and personnel, and the administration route was different between groups, which can easily unseal the blinding |
| Blinding of outcome assessment (detection bias)  All outcomes | Unclear risk | Not reported |
| Incomplete outcome data (attrition bias)  All outcomes | Low risk | No missing data |
| Selective reporting  (reporting bias) | Unclear risk | No protocol was available |
| Other bias | Low risk | No other concerns identified |
